# Supplementary material for: Predicting postoperative delirium assessed by the Nursing Screening Delirium Scale in the recovery room for non-cardiac surgeries without craniotomy: A retrospective study using a machine learning approach
Source: PLOS Digit Health. 2024 Aug 14;3(8):e0000414. doi: 10.1371/journal.pdig.0000414 (PMC11324157; doi:10.1371/journal.pdig.0000414)
Supplement: S2 Appendix — (DOCX) [file pdig.0000414.s002.docx]

### **S2 Appendix**

Table A: Bypassing - and discharge criteria for the recovery room at clinical institution.

| **Criteria for bypassing the recovery room** | **Criteria for recovery room discharge** |
| --- | --- |
| 1. Patients with expected postoperative care >4h and <24h are admitted to the Post Anesthesia Care Unit (PACU) 2. Patients with expected postoperative care >24h are admitted to an Intermediate Intensive Care Unit (MICU) or to an ICU 3. Available beds in the recovery room are managed on a daily basis, direct transfer to ICU might be mandatory due to low capacity | For all patients:   1. Consciousness similar to preoperative status 2. Protective reflexes stable similar to preoperative status 3. Spontaneous breathing similar to preoperative status 4. Stable circulation similar to preoperative status 5. No detected aftereffect of muscle relaxants 6. No significant bleeding 7. Sufficient diuresis 8. Free of pain: NRS <= 3 at rest and NRS <= 5 on stress 9. No hyper- or hypothermia   For outpatients:   1. Drinking without nausea or emesis 2. Spontaneous urination   For spinal anesthesia:   1. Sensor block <1h10 and declining motoric block |

Table B: Baseline characteristics for cohorts meeting inclusion criteria described in the main manuscript. Cohort 1 comprises all adult (≥ 18 years) patients admitted between 2017 and 2020. Cohort 2 includes patients from cohort 1 having non-cardio and non-craniotomy surgical procedures and being admitted to the recovery room. The final cohort is described in Table 1 in the main manuscript.

|  |  | Unit | **Cohort 1** Adult patients admitted 2017 – 2020 | **Cohort 2** Cohort 1 with non-cardio and non-craniotomy admitted to the recovery room |
| --- | --- | --- | --- | --- |
| **Counts** | |  |  |  |
|  | Patients | - | 116,534 | 79,629 |
|  | Stays | - | 150,174 | 89,595 |
|  | Surgeries | - | 172,625 | 96,715 |
|  | Previous admissions | - | 0.98, [0, 0, 1] | 0.94, [0, 0, 1] |
|  | Previous surgeries per stay | - | 1.26, [0, 0, 1] | 1.20, [0, 0, 1] |
| **Demographics** | |  |  |  |
|  | Age | years | 56, [40, 57, 70] | 55, [40, 56, 70] |
|  | Gender | - | 54,771 male (47%)  61,763 female (53%) | 38,267 male (48%)  41,362 female (52%) |
|  | BMI | kg/m² | 31.47, [22.22, 25.19, 28.76] | 26.89, [22.76, 25.67, 29.41] |
|  | ASA status | - | 2.44, [2, 2, 3] | 2.29, [2, 2, 3] |
|  | OP N urgency class | - | 4.31, [4, 5, 5] | 4.09, [3, 5, 5] |
| **Hospitalization** | |  |  |  |
|  | Length of hospital stay | days | 6.43, [2.02, 3.11, 6.00] | 8.73, [2.28, 4.16, 8.43] |
|  | Length of anesthesia | hours | 5.27, [1.04, 1.68, 2.68] | 2.03, [1.13, 1.70, 2.56] |
|  | Length of surgery | hours | 1.41, [0.50, 0.90, 1.59] | 1.18, [0.48, 0.88, 1.53] |
|  | Length of recovery room stay | hours | 2.27, [1.04, 1.68, 2.68] | 1.82, [1.04, 1.54, 2.26] |

Table C: Description of feature encoding and - engineering processes.

| **Feature Domain** | **Time phase** | **Description** |
| --- | --- | --- |
|  |  |  |
| Diagnosis and Comorbidities | TI, T1-T3 | The history of ICD-10 is applied in TI as the number of documentations before the hospital stay. Timestamps for codes are used to identify diagnosis as binary variables during the hospital stay within the corresponding timeline T1-T3. Codes assigned at the end of the hospital stay were not used since they are not available at the prediction time. Variables for cases where no corresponding ICD-10 code was entered into the clinical information system (CIS) are labeled as zero. |
| Hospitalization | TI, T1-T3 | Hospitalization information available at admission like number of previous admissions or emergency admission is assigned to TI. Events like anesthesia, surgery, ICU or recovery room stays are encoded as numeric features holding durations in hours. These durations are calculated using the end timestamp of the corresponding time phase subtracting it with the start timestamp of the event - e.g., for T1 the duration of hospitalization is the difference between anesthesia start and admission timestamp in hours. The number of previous surgeries for a patient’s surgical process and the number of surgeries within one hospital stay are included as well. |
| Demographics | TI | Demographic variables are considered as time-invariant. The domain includes height, age, gender and smoking status. When multiple values are entered for a patient's body length or body height, the median is used to calculate the BMI and store unique values per patient stay. |
| Surgical Procedures | TI | Surgical procedures are documented in German hospitals via the Operationen- und Prozedurenschlüssel (OPS) system which differs from procedure codes in the ICD. Codes are hierarchical organized by affected organ systems or body tracts. These groups like respiratory tract, cardio system or nervous system are used to encode binary time-invariant features for the current surgery as well as previous ones. |
| Scores and Scales | T1-T3 | Critical care severity scores like SOFA or APACHE-II as well as more frequently used scales in a surgical setting e.g. RASS, Pain NRS and ASA status are encoded as discrete time-variant features through T1-T3. The 10th, 50th and 90th percentiles are calculated for each timeline using values from the surgical process per patient and hospitalization to address high, low and moderate levels. |
| Laboratory | T1-T3 | Laboratory values from blood gas analysis or more granular laboratory tests are encoded for each carrier being either blood or urine. Variables such as WBC, pH, bicarbonate or blood urea nitrogen are encoded as time-variant numeric features. Timestamps for the entry point in the CIS are used. For arterial blood gas (ABG) values, the results are available almost immediately due to analysis on the ward. Units are integrated where possible into the International System of Units (SI). The 10th, 50th and 90th percentiles are calculated for each timeline using values from the surgical process per patient and hospitalization to address high, low and moderate levels. |
| Input Classes | T1-T3 | Input classes like antibiotics, benzodiazepines or opioids are constructed with the help of the Anatomical Therapeutic Chemical Classification System (ATC). A binary time-variant variable is assigned to each drug class per patient and surgical process. A value of one indicates that at least one substance from that class was given within the timeframe, zero is assigned to all other cases. |
| Inputs | T1-T3 | The constructed input classes are enhanced with specific frequent drug applications encoded from the application counts exceeding the 90th percentile per drug substance for the defined cohort. The given amount, e.g. for KCl in mmol, the drug volume, e.g., for NaCl in ml or the application rate, e.g., for Propofol in mg/kg/h per patient and per surgical process is considered as a time-variant feature. The 10th, 50th and 90th percentiles are calculated along the sum of amounts and volumes for each timeline using values from the surgical process per patient and hospitalization to address high, low and moderate levels. Missingness for inputs represent a not given or undocumented medication. |
| Outputs | T1-T3 | Outputs like urine spontaneous or blood are measured in ml and time-variant encoded. The 10th, 50th and 90th percentiles are calculated along the sum of volumes for each timeline using values from the surgical process per patient and hospitalization to address high, low and moderate levels. |
| Respiratory | T1-T3 | Respiratory parameters frequently measured or set during anesthesia like FiO2, compliance or peep are coded as time-variant numeric features. The 10th, 50th and 90th percentiles are calculated for each timeline using values from the surgical process per patient and hospitalization to address high, low and moderate levels. |
| Vital Signs | T1-T3 | The vital signs like heart rate, respiratory rate or temperature are coded as time-variant numeric features. The 10th, 50th and 90th percentiles are calculated for each timeline using values from the surgical process per patient and hospitalization to address high, low and moderate levels. |
| EEG Imaging | T1-T3 | Parameters recorded during anesthesia measuring the sedation depth like spectral edge frequencies (SEF) or the patient state index (PSI) are coded as time-variant numeric features. The 10th, 50th and 90th percentiles are calculated for each timeline using values from the surgical process per patient and hospitalization to address high, low and moderate levels. |

### **Cross-Validation on Training Data**

Table D: Hyperparameter descriptions and value ranges used in a 3-fold cross-validation process applied on training data with a multi-layer perceptron model. Gamma was only optimized if a focal loss function was configured, lambda was only included in the search when all available features were used.

| **Hyperparameter** | **Description** | **Values** | **Search Algorithm** |
| --- | --- | --- | --- |
|  |  |  |  |
| n_layers | Number of layers | {4, 8, 12} | Grid search |
| n_nodes | Number of perceptrons per layer | {8, 16, 32, 64, 128} |  |
| loss | Loss function | {bce, focal} |  |
| activation | Activation function | {sigmoid, rectifier} | Random search |
| learn_rate | Learning rate | {1e-2, 1e-3, 1e-4} |  |
| batch_size | Batch size | {64, 128, 256} |  |
| gamma (optional) | Focusing parameter for focal loss | {1, 2, 4} |  |
| lambda (optional) | Regularization parameter for L_1_-norm | {1e-2, 1e-3, 1e-4} |  |

Table E: Evaluation metrics and hyperparameters of MLP models with lowest mean validation loss evaluated with 3-fold cross-validation on initial training set. Sensitivity and specificity are cited for the threshold where their sums maximize, precision is cited with recall > 0.7.

|  | **M1** | **M2** | **M3** | **M12** | **M23** | **M123** |
| --- | --- | --- | --- | --- | --- | --- |
|  |  |  |  |  |  |  |
| **Mean loss** |  |  |  |  |  |  |
| Training | 1.325 | 1.685 | 0.004 | 1.110 | 1.069 | 1.150 |
| Validation | 1.153 | 1.077 | 0.004 | 1.061 | 0.983 | 0.984 |
| **AUROC** |  |  |  |  |  |  |
| Training | 0.745 | 0.811 | 0.818 | 0.835 | 0.857 | 0.882 |
| Validation | 0.721 | 0.775 | 0.791 | 0.782 | 0.823 | 0.822 |
| **AUPRC** |  |  |  |  |  |  |
| Training | 0.263 | 0.337 | 0.350 | 0.375 | 0.433 | 0.469 |
| Validation | 0.242 | 0.295 | 0.291 | 0.303 | 0.375 | 0.372 |
| **Sensitivity** |  |  |  |  |  |  |
| Train | 0.657 | 0.709 | 0.728 | 0.765 | 0.766 | 0.816 |
| Validation | 0.637 | 0.642 | 0.709 | 0.719 | 0.735 | 0.727 |
| **Specificity** |  |  |  |  |  |  |
| Training | 0.698 | 0.754 | 0.760 | 0.745 | 0.784 | 0.790 |
| Validation | 0.688 | 0.769 | 0.742 | 0.699 | 0.766 | 0.769 |
| **Precision** |  |  |  |  |  |  |
| Training | 0.153 | 0.209 | 0.226 | 0.244 | 0.277 | 0.337 |
| Validation | 0.1403 | 0.175 | 0.199 | 0.179 | 0.236 | 0.233 |
| **Feature selection parameters** |  |  |  |  |  |  |
| Missing fraction | 1.00 | 1.00 | 0.40 | 1.00 | 1.00 | 1.00 |
| Numerical threshold | 0.00 | 0.00 | 0.05 | 0.00 | 0.00 | 0.00 |
| Categorical threshold | 0.00 | 0.00 | 0.50 | 0.00 | 0.00 | 0.00 |
| **Hyperparameters** |  |  |  |  |  |  |
| Batch size | 128 | 128 | 64 | 128 | 128 | 256 |
| Loss | bce | bce | focal | bce | bce | bce |
| Activation | rectifier | rectifier | rectifier | rectifier | rectifier | rectifier |
| Learning rate | 1E-4 | 1E-4 | 1E-3 | 1E-4 | 1E-3 | 1E-4 |
| # Nodes | 64 | 128 | 16 | 64 | 128 | 32 |
| # Layers | 4 | 12 | 4 | 4 | 8 | 4 |
| Gamma |  |  | 4 |  | - |  |
| Lamda | 1E-2 | 1E-2 |  | 1E-3 |  | 1E-3 |
| **# of input features** | 195 | 274 | 75 | 441 | 401 | 571 |

Table F: Hyperparameter descriptions and value ranges used in a 3-fold cross-validation process applied on training data with tree-based ensemble models.

| **Hyperparameter** | **Description** | **Values** | **Search Algorithm** |
| --- | --- | --- | --- |
|  |  |  |  |
| tree_type | Type of algorithm | {‘random_forest’, ‘boosted_trees’} | Grid search |
| n_estimators | Number of decision trees | {1, 10, 100, 1000} |  |
| max_depth | Max number of nodes per tree | {2, 4, 8} | Random search |
| min_sample_split | Min number of samples to split a node | {2, 4, 8, 16} |  |
| max_leaf_nodes | Max number of leafs per node | {2, 4, 16, all} |  |

Table G: Evaluation metrics and hyperparameters of tree-based models with lowest mean validation loss evaluated with 3-fold cross-validation on initial training set. Sensitivity and specificity are cited for the threshold where their sums maximize, precision is cited with recall > 0.7.

|  | **M1** | **M2** | **M3** | **M12** | **M23** | **M123** |
| --- | --- | --- | --- | --- | --- | --- |
|  |  |  |  |  |  |  |
| **Mean loss** |  |  |  |  |  |  |
| Training | 0.682 | 0.918 | 0.698 | 1.035 | 0.713 | 0.443 |
| Validation | 0.721 | 0.958 | 0.755 | 1.062 | 0.759 | 0.461 |
| **AUROC** |  |  |  |  |  |  |
| Training | 0.775 | 0.737 | 0.782 | 0.697 | 0.799 | 0.886 |
| Validation | 0.641 | 0.701 | 0.738 | 0.682 | 0.767 | 0.846 |
| **AUPRC** |  |  |  |  |  |  |
| Training | 0.511 | 0.478 | 0.520 | 0.441 | 0.540 | 0.514 |
| Validation | 0.353 | 0.439 | 0.469 | 0.426 | 0.504 | 0.422 |
| **Sensitivity** |  |  |  |  |  |  |
| Train | 0.733 | 0.729 | 0.755 | 0.679 | 0.797 | 0.806 |
| Validation | 0.4874 | 0.663 | 0.676 | 0.654 | 0.741 | 0.768 |
| **Specificity** |  |  |  |  |  |  |
| Training | 0.817 | 0.746 | 0.810 | 0.714 | 0.802 | 0.808 |
| Validation | 0.795 | 0.740 | 0.800 | 0.709 | 0.793 | 0.777 |
| **Precision** |  |  |  |  |  |  |
| Training | 0.267 | 0.206 | 0.264 | 0.082 | 0.266 | 0.357 |
| Validation | 0.082 | 0.082 | 0.126 | 0.082 | 0.245 | 0.276 |
| **Feature selection parameters** |  |  |  |  |  |  |
| Missing fraction | 1 | 0.6 | 0.8 | 0.8 | 1.0 | 1.0 |
| Numerical threshold | 0.00 | 0.05 | 0.10 | 0.05 | 0.00 | 0.00 |
| Categorical threshold | 0.0 | 0.5 | 0.5 | 1.5 | 0.0 | 0.0 |
| **Hyperparameters** |  |  |  |  |  |  |
| Tree type | boosted trees | boosted  trees | random forest | boosted trees | boosted trees | boosted trees |
| N estimators | 1000 | 100 | 100 | 10 | 100 | 100 |
| Max depth | 8 | 4 | 8 | 4 | 4 | 4 |
| Min sample split | 8 | 16 | 16 | 4 | 8 | 16 |
| Max leaf nodes |  |  | 16 | All | All | 4 |
| **# of input features** | 195 | 144 | 50 | 182 | 401 | 571 |

We used Adam [1] as an optimizer for training MLPs. A sigmoid logistic function [2] or a rectifier activation function [3] was chosen for each MLP layer. To avoid overfitting [4], early stopping was implemented, interrupting the training process when the validation loss increased over more than 10 epochs (patience) [5]. For tree based algorithms we also used regularization parameters like max_depth. These were set to prevent the tree structures from being too complex – also called pruning [6]. We used early stopping with patience 10 for boosted trees [7].

### **Final Evaluation**

Table H: Performance metrics for logistic regression (lr) multi-layer perceptron (mlp), and tree-based models (tree) on all training data. Sensitivity and specificity are calculated for the threshold that maximizes their sum. Precision is calculated for the highest threshold for with recall > 0.70. Model variants (M1-M123) consume data from time phases and their combinations T1-T123. Data from TI is included for every model.

|  | Model | AUROC | AUPRC | Sensitivity | Specificity | Precision | F1-Score |
| --- | --- | --- | --- | --- | --- | --- | --- |
| **T1** | |  |  |  |  |  |  |
|  | M1_lr | 0.717 | 0.206 | 0.631 | 0.677 | 0.134 | 0.237 |
|  | M1_mlp | 0.732 | 0.240 | 0.686 | 0.649 | 0.143 | 0.241 |
|  | M1_tree | 0.861 | 0.464 | 0.758 | 0.794 | 0.277 | 0.369 |
| **T2** | |  |  |  |  |  |  |
|  | M2_lr | 0.732 | 0.199 | 0.617 | 0.730 | 0.145 | 0.262 |
|  | M2_mlp | 0.811 | 0.337 | 0.697 | 0.769 | 0.207 | 0.322 |
|  | M2_tree | 0.815 | 0.343 | 0.718 | 0.754 | 0.210 | 0.317 |
| **T3** | |  |  |  |  |  |  |
|  | M3_lr | 0.793 | 0.265 | 0.709 | 0.739 | 0.195 | 0.302 |
|  | M3_mlp | 0.818 | 0.341 | 0.753 | 0.735 | 0.219 | 0.315 |
|  | M3_tree | 0.851 | 0.388 | 0.768 | 0.786 | 0.272 | 0.365 |
| **T12** | |  |  |  |  |  |  |
|  | M12_lr | 0.744 | 0.212 | 0.717 | 0.644 | 0.152 | 0.248 |
|  | M12_mlp | 0.836 | 0.371 | 0.749 | 0.759 | 0.236 | 0.333 |
|  | M12_tree | 0.772 | 0.280 | 0.668 | 0.735 | 0.167 | 0.285 |
| **T23** | |  |  |  |  |  |  |
|  | M23_lr | 0.777 | 0.257 | 0.699 | 0.718 | 0.178 | 0.285 |
|  | M23_mlp | 0.862 | 0.435 | 0.784 | 0.778 | 0.283 | 0.363 |
|  | M23_tree | 0.880 | 0.491 | 0.795 | 0.812 | 0.339 | 0.403 |
| **T123** | |  |  |  |  |  |  |
|  | M123_lr | 0.813 | 0.316 | 0.755 | 0.719 | 0.209 | 0.304 |
|  | M123_mlp | 0.876 | 0.460 | 0.799 | 0.795 | 0.316 | 0.386 |
|  | M123_tree | 0.884 | 0.502 | 0.803 | 0.809 | 0.344 | 0.404 |

Table I: TRIPOD checklist including heading and section references from manuscript. Items relevant only to the development of a prediction model are denoted by D, items relating solely to a validation of a prediction model are denoted by V, and items relating to both are denoted D;V.

| **Section**  **/Topic** | **Item** |  | **Checklist Item** | **Heading / Section** |
| --- | --- | --- | --- | --- |
| **Title and abstract** | | | | |
| Title | 1 / D;V | Identify the study as developing and/or validating a multivariable prediction model, the target population, and the outcome to be predicted. | | Title |
| Abstract | 2 / D;V | Provide a summary of objectives, study design, setting, participants, sample size, predictors, outcome, statistical analysis, results, and conclusions. | | Abstract |
| **Introduction** | | | | |
| Background and objectives | 3a / D;V | Explain the medical context (including whether diagnostic or prognostic) and rationale for developing or validating the multivariable prediction model, including references to existing models. | | Introduction |
|  | 3b / D;V | Specify the objectives, including whether the study describes the development or validation of the model or both. | | Introduction |
| **Methods** | | | | |
| Source of data | 4a / D;V | Describe the study design or source of data (e.g., randomized trial, cohort, or registry data), separately for the development and validation data sets, if applicable. | | Data Extraction and Preprocessing / Appendix 1 |
|  | 4b / D;V | Specify the key study dates, including start of accrual; end of accrual; and, if applicable, end of follow-up. | | Cohort and Target Variable |
| Participants | 5a / D;V | Specify key elements of the study setting (e.g., primary care, secondary care, general population) including number and location of centres. | | Data Extraction and Preprocessing |
|  | 5b / D;V | Describe eligibility criteria for participants. | | Cohort and Target Variable |
|  | 5c / D;V | Give details of treatments received, if relevant. | | Appendix 1 |
| Outcome | 6a / D;V | Clearly define the outcome that is predicted by the prediction model, including how and when assessed. | | Cohort and Target Variable / Perioperative Time Phases |
|  | 6b / D;V | Report any actions to blind assessment of the outcome to be predicted. | | Data Splitting, Cross-Validation and Standardization |
| Predictors | 7a / D;V | Clearly define all predictors used in developing or validating the multivariable prediction model, including how and when they were measured. | | Data Extraction and Preprocessing /  Appendix1 and 2 |
|  | 7b / D;V | Report any actions to blind assessment of predictors for the outcome and other predictors. | | N/A |
| Sample size | 8 / D;V | Explain how the study size was arrived at. | | Cohort and Target Variable |
| Missing data | 9 / D;V | Describe how missing data were handled (e.g., complete-case analysis, single imputation, multiple imputation) with details of any imputation method. | | Data Splitting, Cross-Validation and Standardization |
| Statistical analysis methods | 10a / D | Describe how predictors were handled in the analyses. | | Cohort and Target Variable / Perioperative Time Phases |
|  | 10b / D | Specify type of model, all model-building procedures (including any predictor selection), and method for internal validation. | | Data Splitting, Cross-Validation and Standardization / Machine Learning Techniques and Hyperparameter Search / Appendix 2 |
|  | 10c / V | For validation, describe how the predictions were calculated. | | Evaluation Metrics / Model Evaluation |
|  | 10d / D;V | Specify all measures used to assess model performance and, if relevant, to compare multiple models. | | Evaluation Metrics / Model Evaluation |
|  | 10e / V | Describe any model updating (e.g., recalibration) arising from the validation, if done. | | Machine Learning Techniques and Hyperparameter Search / Appendix 2 |
| Risk groups | 11 / D;V | Provide details on how risk groups were created, if done. | | N/A |
| Development vs. validation | 12 / V | For validation, identify any differences from the development data in setting, eligibility criteria, outcome, and predictors. | | Appendix 1 |
| **Results** | | | | |
| Participants | 13a / D;V | Describe the flow of participants through the study, including the number of participants with and without the outcome and, if applicable, a summary of the follow-up time. A diagram may be helpful. | | Cohort and Target Variable |
|  | 13b / D;V | Describe the characteristics of the participants (basic demographics, clinical features, available predictors), including the number of participants with missing data for predictors and outcome. | | Cohort and Target Variable / Appendix 1 |
|  | 13c / V | For validation, show a comparison with the development data of the distribution of important variables (demographics, predictors and outcome). | | Cohort and Target Variable / Appendix 1 |
| Model development | 14a / D | Specify the number of participants and outcome events in each analysis. | | Cohort and Target Variable |
|  | 14b / D | If done, report the unadjusted association between each candidate predictor and outcome. | | Perioperative Variables / GitHub |
| Model specification | 15a / D | Present the full prediction model to allow predictions for individuals (i.e., all regression coefficients, and model intercept or baseline survival at a given time point). | | Appendix 2 / GitHub |
|  | 15b / D | Explain how to the use the prediction model. | | GitHub |
| Model performance | 16 / D;V | Report performance measures (with CIs) for the prediction model. | | Model Evaluation |
| Model-updating | 17 / V | If done, report the results from any model updating (i.e., model specification, model performance). | | Appendix 2 |
| **Discussion** | | | | |
| Limitations | 18 / D;V | Discuss any limitations of the study (such as nonrepresentative sample, few events per predictor, missing data). | | Limitations |
| Interpretation | 19a / V | For validation, discuss the results with reference to performance in the development data, and any other validation data. | | Model Evaluation |
|  | 19b / D;V | Give an overall interpretation of the results, considering objectives, limitations, results from similar studies, and other relevant evidence. | | Comparison with Related Work / Limitations |
| Implications | 20 / D;V | Discuss the potential clinical use of the model and implications for future research. | | Clinical Interpretation / Limitations / Conclusion |
| **Other information** | | | | |
| Supplementary information | 21 / D;V | Provide information about the availability of supplementary resources, such as study protocol, Web calculator, and data sets. | | Code Availability and Reporting |
| Funding | 22 / D;V | Give the source of funding and the role of the funders for the present study. | | Funding Source |

Fig A: Distribution of durations between one event and another one during the perioperative phase. Histogram is stacked summing up counts from POD positive (y=1) and negative (y=0) groups.


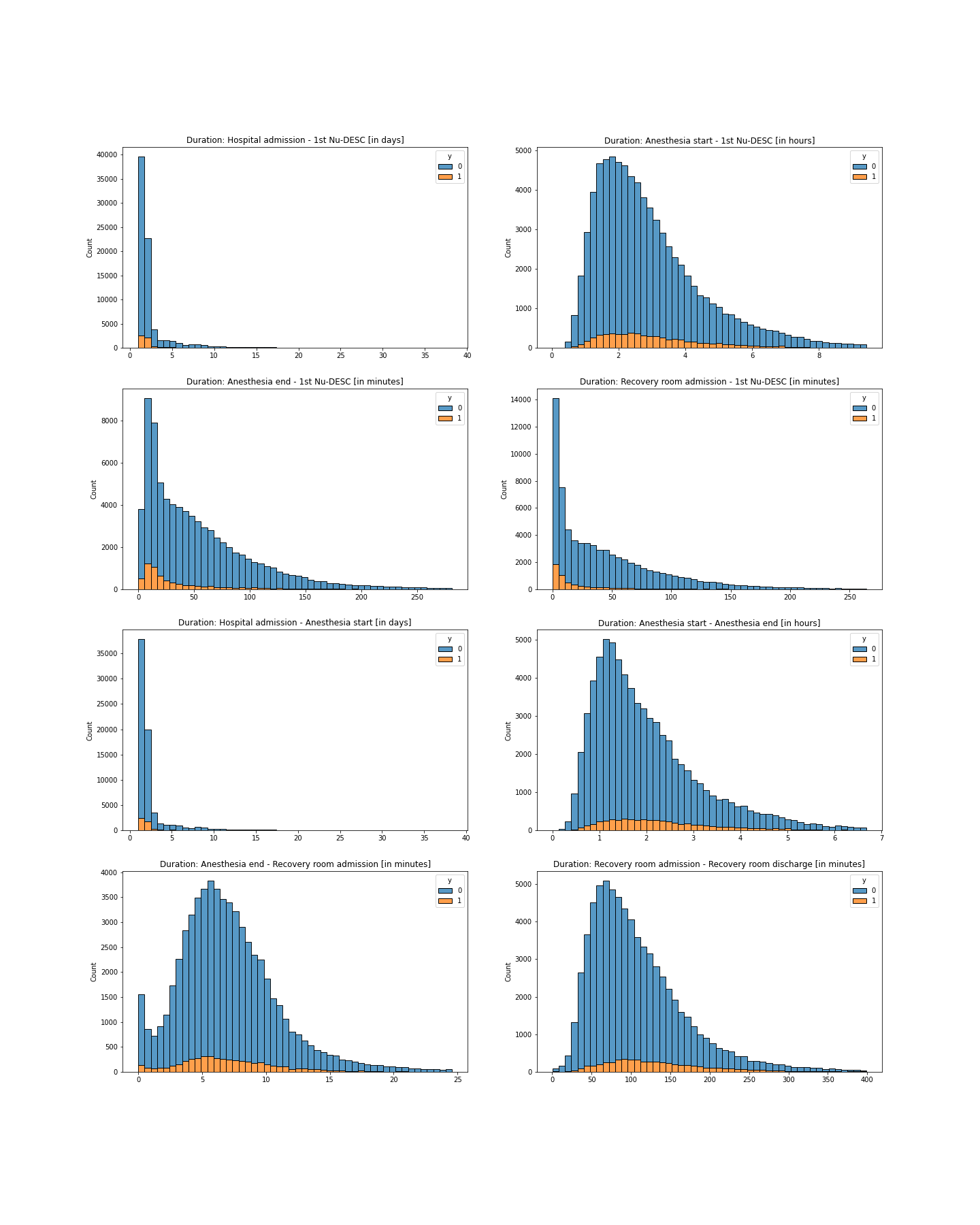


Fig B: Pearson correlation coefficient for binary missing indicator variables per feature. Top 10 features are shown according to their highest sum of absolute coefficient to all other variables.


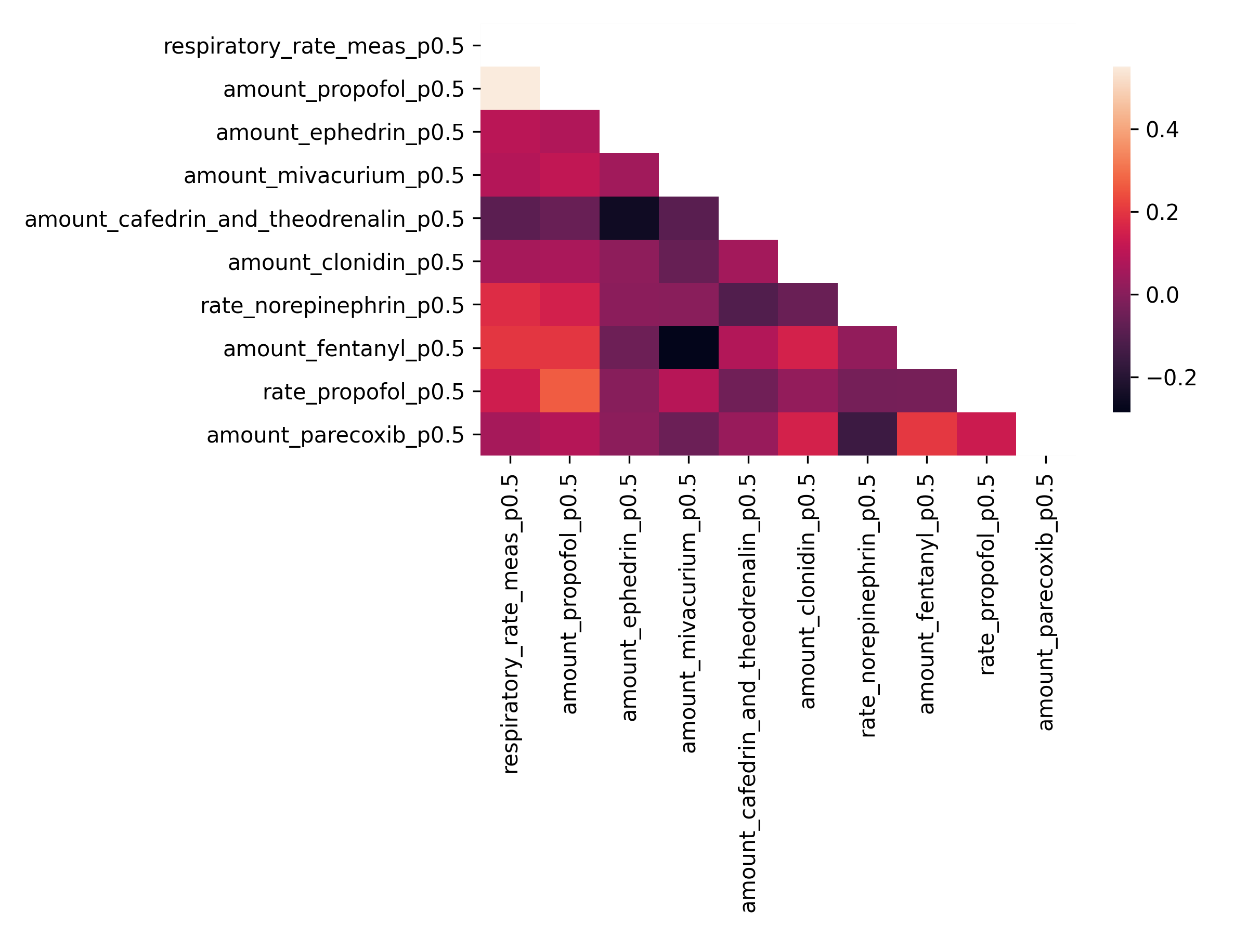


We investigated the correlation between feature missing indicator variables. Fig B presents Pearson correlation coefficients [8] (c) calculated between these indicator variables. We observed a relatively high correlations between the missingness of given amounts and rates of medications, e.g., amounts fentanyl and propofol (c=0.29). The absence of the hypnotic drug propofol and the respiratory rate for intubated breathing patients had the highest correlation (c=0.48). This relationship can be explained by the induction of sedation where the presence of propofol also results in the presence of respiratory rates recordings that are set for the ventilator [9].

Fig C: Area under the receiver operating characteristics curve (AUROC) calculated on the test set per time phase combination (T1-T123). Referenced baseline models are indicated by 1st author’s name – Wassenaar or Boogaard - as prefix, recalibrated models are models are indicated as rec. Baseline models were either pre-trained (pretr), retrained with a logistic regression - (lr) or a multi-layer perceptron (mlp) approach.


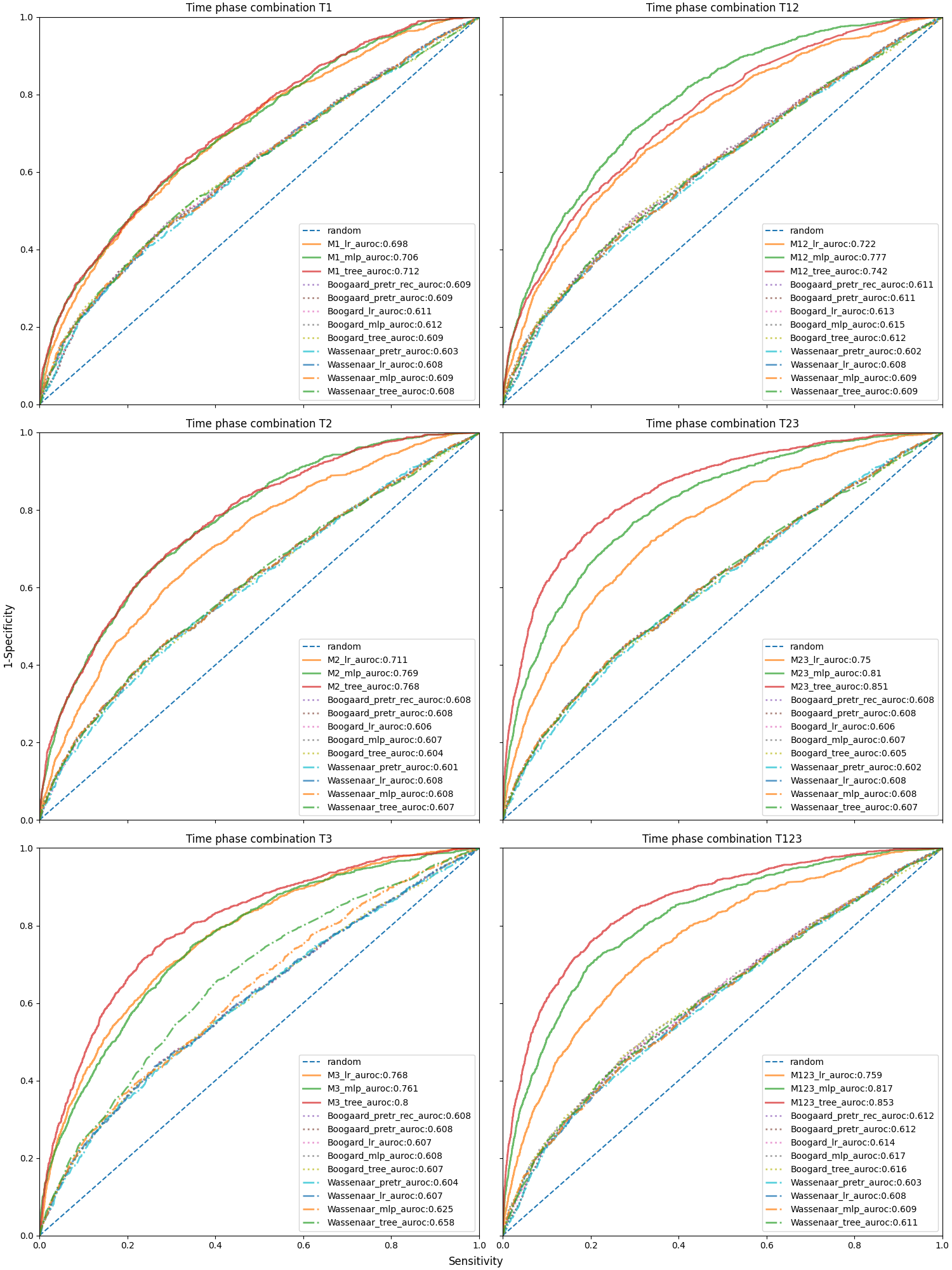


Fig D: Area under the precision-recall curve (AUPRC) calculated on the test set per time phase combination (T1-T123). Referenced baseline models are indicated by 1st author’s name – Wassenaar or Boogaard - as prefix, recalibrated models are models are indicated as rec. Baseline models were either pre-trained (pretr), retrained with a logistic regression - (lr) or a multi-layer perceptron (mlp) approach.


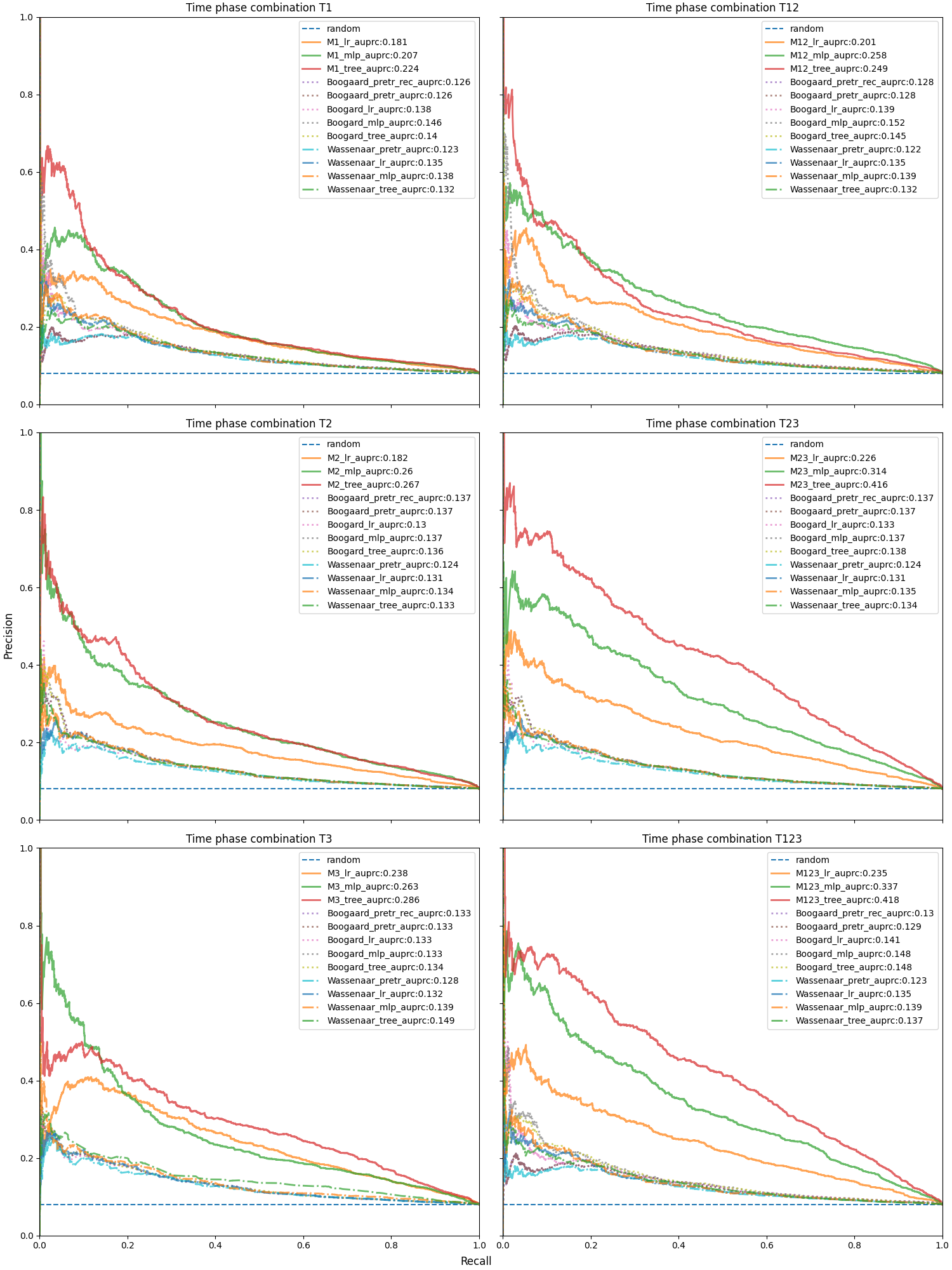


Table J: Brier calibration scores as mean squared error between predicted probabilities and true labels. Scores are ordered by model type (columns) and included perioperative phases (rows).

|  | **MLP** | **Tree-based** |
| --- | --- | --- |
| **T1** | 0.135 | 0.177 |
| **T2** | 0.131 | 0.176 |
| **T3** | 0.215 | 0.197 |
| **T12** | 0.170 | 0.191 |
| **T23** | 0.130 | 0.146 |
| **T123** | 0.099 | 0.143 |

Fig E: Calibration plots showing the fraction of positive cases and the mean predicted probability for MLP models.


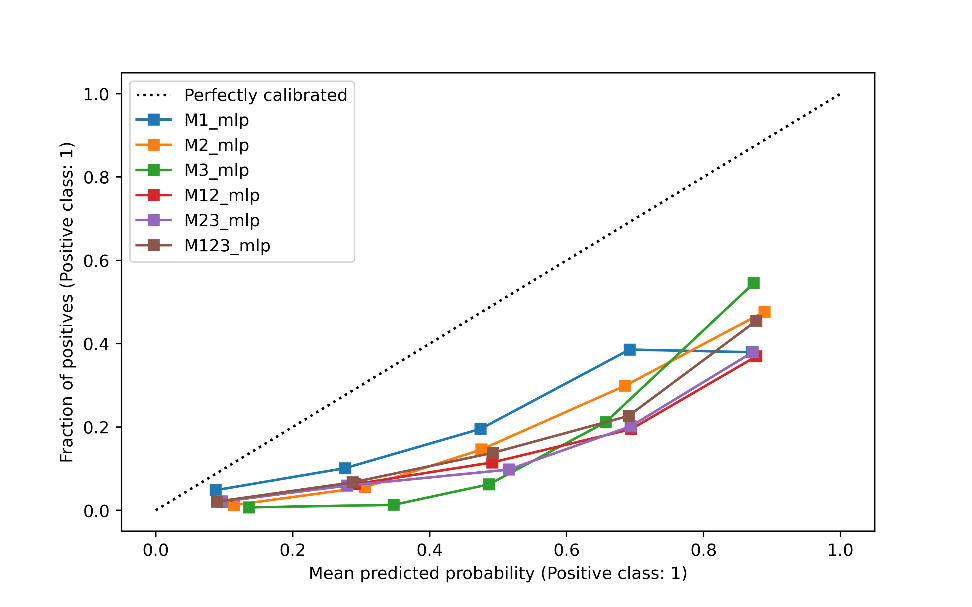


Fig F: Calibration plots showing the fraction of positive cases and the mean predicted probability for tree models.


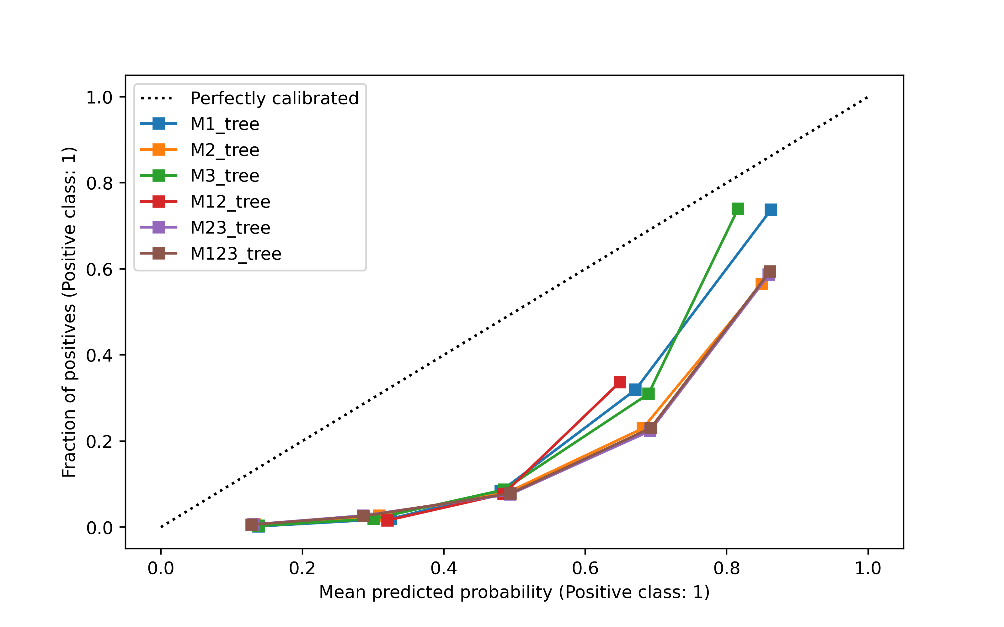


### **References**

1. Kingma, D. P., & Ba, J. (2014). Adam: A method for stochastic optimization. arXiv preprint arXiv:1412.6980.
2. Verhulst, P. F. (1838). Notice sur la loi que la population suit dans son accroissement. Corresp. Math. Phys., 10, 113-126.
3. Fukushima, K. (1969). Visual feature extraction by a multilayered network of analog threshold elements. IEEE Transactions on Systems Science and Cybernetics, 5(4), 322-333.
4. Hawkins, D. M. (2004). The problem of overfitting. Journal of chemical information and computer sciences, 44(1), 1-12.
5. Prechelt, L. (1998). Automatic early stopping using cross validation: quantifying the criteria. Neural networks, 11(4), 761-767.
6. Mingers, J. (1989). An empirical comparison of pruning methods for decision tree induction. Machine learning, 4(2), 227-243.
7. Zhang, T., & Yu, B. (2005). Boosting with early stopping: Convergence and consistency. The Annals of Statistics, 33(4), 1538-1579.
8. Sedgwick, P. (2012). Pearson’s correlate on coefficient. Bmj, 345.
9. Tobin, M. J., Laghi, F., & Jubran, A. (2012). Ventilatory failure, ventilator support, and ventilator weaning. Comprehensive Physiology, 2(4), 2871-2921.
