# Supplementary material for: Predicting postoperative delirium assessed by the Nursing Screening Delirium Scale in the recovery room for non-cardiac surgeries without craniotomy: A retrospective study using a machine learning approach
Source: PLOS Digit Health. 2024 Aug 14;3(8):e0000414. doi: 10.1371/journal.pdig.0000414 (PMC11324157; doi:10.1371/journal.pdig.0000414)
Supplement: Attachment — Submitted filename: reviewers_comments_17_05_2024.docx [file pdig.0000414.s003.docx]

Reviewer's Responses to Questions

<font color="black"><b>Comments to the Author</b><br><br>

1. Does this manuscript meet PLOS Digital Health’s <a href="https://journals.plos.org/digitalhealth/s/journal-information#loc-criteria-for-publication" target="_blank">publication criteria</a>? Is the manuscript technically sound, and do the data support the conclusions? The manuscript must describe methodologically and ethically rigorous research with conclusions that are appropriately drawn based on the data presented.</font>

Reviewer #1: Yes

Reviewer #2: Yes

Reviewer #3: No

RESPONSE: We present results drawn by implementing robust statistical testing methods as well as sophisticated machine learning models. Firstly, we presented univariate statistical results to describe the discriminative power of single clinical variables towards our POD endpoint. Afterwards, we selected specific feature subsets based on these univariate results and additionally included all available features. By using L_1_ regularization and weighted loss techniques, we increased the robustness and applicability of our models. Finally, we performed an uncertainty estimation by calculating confidence intervals on resampled testing sets.

In this revision, we have extended our descriptive statistics for extracted and aggregated Features in Appendix 1 in Extended Tables 3 and 4. Moreover, we have uploaded all these data including cohort specific information on the public data repository Dryad as suggested by PLOS Digital Health. Data are available for direct download under <https://datadryad.org/stash/share/JDGY_rqv4nmQodtW0FX5UOzEv93726JABMa0nVB2ZbM>.

--------------------

<font color="black">2. Has the statistical analysis been performed appropriately and rigorously?</font>

Reviewer #1: I don't know

Reviewer #2: Yes

Reviewer #3: No

RESPONSE: We present univariate statistical results from robust Mann-Whitney U (MWU) statistics. These statistics are non-parametric, meaning that they do not assume that underlying values are normally distributed. Hence, we found the MWU test as appropriate for analyzing clinical data records that could potentially include outliers or follow skewed distributions. We adjusted resulting p-values with false discovery rate (FDR) correction to mitigate Type I (false positive) errors that occur when the null-hypothesis was falsely rejected.

Machine learning models were trained on independent train- and testing sets. For the comparison of model performances, we applied models on a 1000 x bootstrapped testing set. Retrieving multiple performance scores on different resampled testing sets enabled us to estimate the stability of our models by calculating confidence intervals (CI). Our statistical analysis plan and its execution was discussed with a senior statistician and deemed appropriate.

We highlighted these aspects in the main manuscript by adding “FDR correction mitigates Type I (false-positive) errors that occur when falsely rejecting the null-hypothesis for multiple testing problems” and “By applying bootstrapping, we sought to estimate the stability of model predictions.”

--------------------

<font color = "black">3. Have the authors made all data underlying the findings in their manuscript fully available (please refer to the Data Availability Statement at the start of the manuscript PDF file)?<br><br> The <a href="https://journals.plos.org/digitalhealth/s/data-availability" target="_blank">PLOS Data policy</a> requires authors to make all data underlying the findings described in their manuscript fully available without restriction, with rare exception. The data should be provided as part of the manuscript or its supporting information, or deposited to a public repository. For example, in addition to summary statistics, the data points behind means, medians and variance measures should be available. If there are restrictions on publicly sharing data—e.g. participant privacy or use of data from a third party—those must be specified.</font>

Reviewer #1: No

Reviewer #2: Yes

Reviewer #3: No

RESPONSE: We uploaded descriptive data and enhanced the Code- and Data Availability, Reporting section.

“The code including trained models, preprocessing scripts and, usage notes, and descriptions are openly accessible on our public code repository (55). We published descriptive data for our training- and testing sets on a public Dryad repository(56). We foster the reproducibility of our results by describing our data preprocessing pipeline and by providing guidance for applying our models. German data protection rules - that are based on the European General Data Protection Regulation (GDPR) - prohibit publicly sharing original patient data such as ours (57). However, we present descriptive statistics of extracted and aggregated EHR in Extended Table 3 and 4 in Appendix 1.”

Data are available for direct download under the public repository Dryad suggested by PLOS Digital Health <https://datadryad.org/stash/share/JDGY_rqv4nmQodtW0FX5UOzEv93726JABMa0nVB2ZbM>.

--------------------

<font color = "black">4. Is the manuscript presented in an intelligible fashion and written in standard English?<br/><br/>PLOS Digital Health does not copyedit accepted manuscripts, so the language in submitted articles must be clear, correct, and unambiguous. Any typographical or grammatical errors should be corrected at revision, so please note any specific errors here.</font>

Reviewer #1: Yes

Reviewer #2: Yes

Reviewer #3: No

RESPONSE: The manuscript underwent multiple rounds of proofreading before submission. We do not believe that our manuscript suffers from typographical or grammatical errors. We thank Reviewer #3 for suggesting a few rewordings, which we have all included in the revised version of our manuscript.

Specifically, we have rephrased the following sections:

“Significant univariate impact factors were the preoperative ASA status (American Society of Anesthesiologists physical status classification system), the intraoperative amount of given remifentanil, and the postoperative Aldrete score.” (Abstract)

“Many patients who experience POD suffer from chronic mental disorders causing pressure on related family members, clinicians, and the health system. With our study, we want to detect suspected POD before onset trying to give decision support to health professionals. Vulnerable patients could be transferred to a higher level of care mitigating the risk of severe outcomes such as long-term cognitive decline.” (Introduction)

--------------------

<font color = "black">5. Review Comments to the Author<br/><br/>Please use the space provided to explain your answers to the questions above. You may also include additional comments for the author, including concerns about dual publication, research ethics, or publication ethics. (Please upload your review as an attachment if it exceeds 20,000 characters)</font>

**Reviewer #1:**

In the manuscript "Predicting postoperative delirium in the recovery room", the authors constructed a predictive model for postoperative delirium using clinical information from a large cohort of patients. I have several issues:

1. Did any patients in the study undergo multiple surgeries during the observation period? For such patients, were all surgical records included in the study, or only records from the initial surgery?

RESPONSE: Thank you for your comment. We found multiple surgeries for one single patient during the observation period and included all surgeries in our analysis. According to your comment, we added “Each POD target variable y was calculated per surgery directly corresponding to the hospital stay and the admitted patient. Multiple surgeries during one hospitalization were treated as individual samples.” in the cohort and target variable section. Please also find summary statistics as the number of surgeries per hospital stay and the number of previous surgeries for one stay and one patient in Table 1.

2. The appendix mentions,“A feature selection algorithm was devised to provide a tradeoff between data availability and predictive power towards the target.” Is this algorithm designed for variable selection? Or are you including all variables as predictor variables in the model? If feature selection was performed, please provide a detailed explanation of the selection method and the predictor factors ultimately included in the models.

RESPONSE: Thank you for your valuable question. We agree that our feature selection algorithm for selecting variables, as you mentioned, should be explained in the main manuscript. We extended the Univariate Test Statistics section by details on our feature selection algorithm and the input features of our model variants. We introduced two different feature selection methods. One method based on univariate statistics heuristically selecting subsets of features. The other method included all features and actively selected predictors (also see answer to question 7) in a multivariable approach.

“Robust Mann-Whitney U (MWU) tests (38)[31] were used to evaluate the discriminatory power of numerical parameters with respect to the POD target. We applied false discovery rate (FDR) correction (39)[32] with (alpha=0.05) was applied to identify statistically significant variables. FDR correction mitigates Type I (false-positive) errors that occur when falsely rejecting the null-hypothesis for multiple testing problems (40). The AUROC – which can be derived from the MWU statistic (41)[33] – was used to quantify effect-sizes. AUROC values are normalized between 0 and 1, where 1 indicates a perfect positive association, 0 indicates a perfect negative association, and 0.5 indicates chance-level discrimination. The absolute strength of an effect – regardless of the direction – was calculated for each significant variable as e=2|AUROC-0.5|. For categorical variables, we used the odds ratio (OR) of a univariate logistic regression (42)[34] as a measure of effect size. A direction independent measure of effect size was defined as o=|log(OR)|.

We applied two different methods to select model input features out of 375 variables that were available for at least 10% of the patients. The first method devised a feature selection algorithm to provide a tradeoff between data availability and predictive power towards the target. For numerical features, missingness (m) was furthermore defined as the fraction of patients having no values for more than 50% of all available features. As a measurement for the univariate predictive performance, the previously defined parameters e and o were used for numerical and categorical variables respectively. A grid search (43) was applied over different thresholds for m (tm = [0.4, 0.6, 0.8]), e (te = [0.1, 0.05]) and o (to = [1.5, 0.5]).

Numerical features were ordered descending by their univariate performance parameter *e*. A numerical feature was drawn and added to a feature set if its value for e exceeded the threshold *te* and if the missingness in the set did not exceed the threshold *tm*. Categorical features were simply added if their values for *o* were above the corresponding threshold *to*. When multiple time phases (T1-T3) were included for a model variant, the mean of *e, o*, and *m* was calculated across these phases. In case of multiple summary statistics (e.g., percentiles) for the same feature, only the most discriminative one was ultimately included in the feature set.

The second feature selection method was performed with all available 375 features (tm = 1, te = 0, to = 0). We applied L1-norm regularization (44) to automatically detect input variables that contribute to the POD prediction task. In contrast to the first univariate selection method, we took inter-correlation between covariates into account here. Models predicted our target and selected features in parallel. In total, we run experiments with 3 * 2 * 2 + 1 = 13 feature sets per model variant (M1-M123) including 12 sets from the first selection method and one set from the second method. The loss calculated on a validation set was used as a cost function.”

Extended Table 3 in Appendix 1 hold descriptive statistics of extracted features. We aggregated multiple feature values per surgery with percentiles and sums. Extended Table 4 in Appendix 1 outline statistics for these aggregations. A list of all input features per model are included in Extended Table 5 in Appendix 1.

3. In the methods section，it mentions“To avoid dependencies between these sets we used patient identifiers to perform the splitting. Stratification with the target variable was done so that the incidence of POD was preserved in both sets.” Was the training set and validation set not divided randomly? Were there differences in the variables between the training set and validation set？

RESPONSE: Thank you for the comment. As you pointed out, we randomly split our data in training and testing sets based on patient identifiers. Hereby, we could maintain independence between these sets for unbiased predictions. We rephrased our sentence with “The extracted data were initially randomly split (80/20%) into train – and test sets.” We provide insights into statistics for comparing variables in training as well as validation sets. We clarified this by adding “In Extended Table 3 in Appendix 1, we present descriptive statistics for both data sets indicating that the test set pose a representative sample of our training set.”

Additionally, we explained how we imputed missing values with “Extracted training set mean values were also used to impute missing values in train, validation, and test sets. Mean imputation was applied as calculating the mean of all values per feature across the population and replacing missing values by these feature means.”

4. In the methods section，it mentions “Extracted training set mean values were also used to impute missing values in train, validation, and test sets.” What is the reason for having three datasets?

RESPONSE: Thank you for this remark. For searching ML model configurations, the training data are usually split into further training and validation sets [1]. Since models include parameters that are optimized during training, and hyperparameters that are chosen prior to training, it is customary to perform the additional split to "test" for an optimal choice of hyperparameters on the hold-out (validation) splits. After finding the best configurations of hyperparameters, we retrained models and applied them to the final testing set.

We made our sentence more clear by rephrasing “A 3-fold cross validation (CV) technique was applied where a hyperparameter configuration was determined from 66.6% (2 training folds) and evaluated on 33.3% (1 hold-out fold) of the training data”. Moreover, we added “Feature mean values as well as standard deviations were calculated on the training data, applied to validation (hold-out folds) – and eventually to the test data.”

[1] Stone M. Cross-Validatory Choice and Assessment of Statistical Predictions. J R Stat Soc Ser B Stat Methodol. 1974 Jan 1;36(2):111–33.

5.In the table 3, is the definition of the missing rate accurate? And in the addendix, “all numerical features that were not available for at least 10% of the patients were dropped”, however, there are many features in the table3 with a missing rate higher than 10%, please explain this. Additionally, would the presence of numerous features with high missing rates affect the model's outcomes? Would it compromise the credibility of the model?

RESPONSE: Thank you for sharing this sharp observation. We initially extracted more features (exceeding 90% missingness) for perioperative time phases than we eventually selected for our models. According to your comment, we changed our Feature Extraction and Preprocessing section as “Based on literature review and clinical expertise, a total of 549 clinical variables were identified with respect to T1-T3 and TI in our source systems. From these variables, 375 were available for at least 10% of the included patients.” Extended Table 3 in Appendix 2 lists all 375 variables increasing consistency. Our feature selection algorithm systematically investigated features with less than 90% missingness and combined them into sets for a Grid-Search process. Selected features per model variant are outlined in Extended Table 5 in Appendix 1.

In the discussion, we added “Some features also showed high predictive power but low availability. We encoded feature availability information as missing indicators that could be learned.” We believe that our models could benefit from rarely documented but strongly discriminative features. Our additional analysis reading model weights for input features could support this assumption since it highlighted binary missing indicators as predictive variables (see response to question 8).

6.In table 3, “Highly correlated clinical variables were age (e=0.232, with e=2|AUROC-0.5|) for TI, the ASA status (e=0.179) for T1, the intraoperative (T2) amount of remifentanil (e=0.200), and the Aldrete score (e=0.347) measured in the recovery room (T3).”Why isn't it Anesthesia duration (e=0.218)? This seems inconsistent with the results in Table 3.

RESPONSE: Thank you for your comment. You are right with identifying anesthesia duration as highly correlated with POD. We wanted to highlight the importance of duration information for the discrimination of POD in a separate sentence. We described results in more depth by adding “The anesthesia-, and the surgery durations calculated for each timeline are highly discriminative in both, the intraoperative - (T2) (anesthesia duration e=0.218, surgery duration e=0.183) and the postoperative (T3) (anesthesia duration e=0.219, surgery duration e= 0.183) phase.”

7. please provide a detailed explanation of the predictor variables in the best model

RESPONSE: Thank you for requesting more information about predictor variables that were important for our machine learning models. Besides listing input variables in Extended Table 5 of Appendix 1, we extracted model weights for features that were important for our best applicable MLP model M12. We added “Additionally, we read MLP weights from the very first layer to give insights into input features that were focused during learning.” in the section Machine Learning Techniques and Hyperparameter Search.

Furthermore, we added “We retrieved MLP input weights (w) for 441 features including missing indicators recorded for the time phase T12. We found age (w=0.907), history of delirium (w=0.784), and history of hypertension (w=0.631) as important model inputs. These variables describe predisposing factors that might contribute to illness severity increasing the chance of POD. We observed elevated model weights regarding the binary missing indicator for intraoperative pulse (w=0.526) and sedation depth (w=0.379). Observations suggest that POD predictions can benefit from encoded availability information. The median-aggregated amount of remifentanil given during the intraoperative phase (T2) yielded a weight of 0.337. The predictor showed a positive POD correlation in univariate analyses in line with guidelines that suggest opioid sparse sedations. In Extended Table 6 in Appendix 1, we present further model weights. Results were complementary with univariate findings.”

8. The authors need to adhere to STROBE guidelines. There are numerous other requirements for adequate reporting of a study.

RESPONSE: Thank you for your remark. We agree that we need to adhere to reporting guidelines. To our knowledge, STROBE [2] guidelines are designed for observational studies in epidemiology. Since we conducted a retrospective study using machine learning models with electronic health records, we report results in accordance with the transparent reporting of a multivariable prediction model for individual prognosis or diagnosis (TRIPOD) [3] guidelines (see Extended Table 10 in Appendix 2).

[2] Cuschieri, S. (2019). The STROBE guidelines. Saudi journal of anaesthesia, 13(Suppl 1), S31-S34.

[3] Patzer, R. E., Kaji, A. H., & Fong, Y. (2021). TRIPOD reporting guidelines for diagnostic and prognostic studies. JAMA surgery, 156(7), 675-676.

9. Please specify the strengths of this study in the discussion section.

RESPONSE: Thank you for suggesting including strengths in the discussion section. According to your comment, we added a separate paragraph in our Discussion.

“We systematically investigated the contribution of aggregated clinical variables to the prediction of POD under varying perioperative time phases. The combination of features from multiple time phases improved predictions made by highly non-linear machine learning models. We extracted model weights for providing insights into our best performing applicable MLP model M12. Results were accompanied by robust univariate test statistics revealing effect sizes as well as effect directions towards our POD target variable. Our models are openly accessible and can be evaluated in other medical centers.”

Reviewer #2: This study significantly contributes to the field by applying ML to predict POD in a recovery room setting. The methodology is rigorous, and the findings have clear implications for improving patient outcomes post-surgery. Including pre-, intra-, and postoperative data in the ML models is a notable strength. The paper dealt with rare events. The author used specific metrics for rare event prediction, and both AUROC and AUPRC metrics thoroughly evaluate model performance. However, the paper could be strengthened in the following aspects:

1. The introduction might be enhanced by more explicitly stating the study’s novel contributions beyond the application of ML, particularly how it advances understanding of POD risk factors or patient outcomes.

RESPONSE: Thank you for suggesting enhancing our introduction by novel aspects. According to your suggestion, we added “The application of prognostic models was accompanied by analyses of effect sizes and effect directions for single predictors on POD to understand risk factors in more depth.” to our Introduction.

2. While the methods are generally strong, the paper could provide more detail on handling missing data and justifying the choice of ML models over others.

RESPONSE: Thank you for requesting additional information about handling missing data and the choice of ML models. We added “Extracted training set mean values were also used to impute missing values in train, validation, and test sets. Mean imputation was applied as calculating the mean of all values per feature across the population and replacing missing values by these feature means.” to the Data Splitting, Cross-Validation and Standardization section. Moreover, we added “A binary indicator variable was added to encode real missingness of numerical variables” to the Feature Encoding section.

We justified our selection of ML methods by formulating “Previous studies have shown that MLPs can describe highly complex non-linear functions acting as universal approximators.” and “We selected additional ensemble techniques due to their capability of separately learning aspects of the training data and combining results into one final prediction”.

3. Would over-sample or down-sample further help predict the “rare event”?

RESPONSE: We think that over- or under sampling techniques, like SMOTE [1], would potentially bias our data and do not reflect the true observed incidence. For addressing class imbalance, we implemented a weighted loss function and described, “We used focal loss or weighted binary cross-entropy (BCE) since they have been shown to be able to deal with unbalanced classification problems such as ours.”

[1] Elreedy, D., & Atiya, A. F. (2019). A comprehensive analysis of synthetic minority oversampling technique (SMOTE) for handling class imbalance. *Information Sciences*, *505*, 32-64. <https://doi.org/10.1016/j.ins.2019.07.070>

4. Which tree method gave the best performance? Random forest or boosting trees?

RESPONSE: Thank you for your remark. We specified the best performing tree models by adding “The non-linear boosted trees model achieved a mean AUROC of 0.854 and a mean AUPRC of 0.418 outperforming linear LR, well as best applied and retrained baseline models.” in the Abstract.

Furthermore, we integrated “The best performing non-linear boosted trees model variant M123 was trained on all perioperative data (T123). This model showed a mean AUROC of 0.854 (95% CI [0.853, 0.855]) and a mean AUPRC of 0.418(95% CI [0.415, 0.421]]).” in the Results section.

Additionally, we presented model configurations and wrote, “Extended Table 8 in Appendix 2 shows training results for tree based models. Gradient boosted trees were evaluated as superior to random forest during all perioperative phases except for the single postoperative phase (T3).” in the Results section.

5. The study's single-center nature may limit the generalizability of the findings. Is it possible to find an external validation cohort?

RESPONSE: Thank you for suggesting external validation. We agree that an external validation cohort would prove the generalizability of our trained models. Unfortunately, we could neither find a publicly available dataset nor an additional protected dataset to validate our results. Other clinical centers in Germany were not willing to share data due to strict privacy protection regulations.

However, we included various multiple strategies to mitigate overfitting to our single-center data set, e.g., L_1_ regularization, bootstrapping, patient specific train/test split.We also agree that this is a limitation and included the following paragraph: “Many openly accessible clinical research databases comprise ICU data instead of perioperative clinical records hampering validation of our results. We also limited our target variable to Nu-DESC observations that might not be available in other clinical centers.”

6. The results section could benefit from a deeper analysis of which specific features contributed most to the prediction models and why.

RESPONSE: We totally agree with your suggestion explaining predictor variables in more depth. Thus, we wrote a novel paragraph in our Discussion.

“We retrieved MLP input weights (w) for 441 features including missing indicators recorded for the time phase T12. We identified age (w=0.907), history of delirium (w=0.784), and history of hypertension (w=0.631) as important model inputs. These variables describe predisposing factors that might contribute to illness severity increasing the chance of POD. We observed elevated model weights regarding the binary missing indicator for intraoperative pulse (w=0.526) and sedation depth (w=0.379). Observations suggest that POD predictions can benefit from encoded availability information. The median-aggregated amount of remifentanil given during the intraoperative phase (T2) yielded a weight of 0.337. The predictor showed a positive POD correlation in univariate analyses in line with guidelines that suggest opioid sparse sedations. In Extended Table 6 in Appendix 1, we present further model weights. Results were complementary with univariate findings.”

7. Additionally, discussing the clinical relevance of prediction accuracy in practical settings would be beneficial.

RESPONSE: Thank you for highlighting the importance of discussing results in a concrete clinical setting. We provided information on a potential model implementation.

“In clinical practice, it is desirable to know the risk for POD at the end of the intraoperative phase. This knowledge could be used to initiate preventive measures such as transportation to a noise-reduced ward after the surgery. Models ingesting T12 make predictions before the admission to a recovery room. Thus, the physician can decide to transfer the patient to a specialized ward.

Assuming 100 surgeries per day through all three hospital sites including 10 real cases of POD. The application of the MLP M12 with a fixed sensitivity at 0.80 and a corresponding precision of 0.20 would lead to 8 correct transfers – of patients really suffering from POD – and 32 incorrect transfers – of patients not suffering from POD – to a specialized ward after surgical procedures. With a usual ICU size of 15-20 patients, the results highlight that a low precision is a main impediment for implementing trained models in a real clinical setting. We provide a configurable prediction model for balancing overtreatment and patient safety.”

8. Calibration metrics were not provided.

RESPONSE: Thank you for pointing out calibration metrics. We calculated Brier [2] scores as they describe the mean squared error between predicted probabilities and true labels. We also plotted calibration plots showing the fraction of positive cases and the mean predicted probability. Please find corresponding material in Extended Table 10, in Extended Figure 5, and in Extended Figure 5 as part of the Appendix 2.

[2] Rufibach, K. (2010). Use of Brier score to assess binary predictions. Journal of clinical epidemiology, 63(8), 938-939.

9. The discussion of limitations is brief. A more detailed exploration of the study's limitations, including its retrospective design and potential for bias, would strengthen the paper.

RESPONSE: We totally agree that explaining limitations should be a central part of our manuscript. Thus, we extended our discussion with “Many openly accessible clinical research databases comprise ICU data instead of perioperative clinical records hampering the validation of our results. We also limited our target variable to Nu-DESC observations that might not be available in other clinical centers. Our cohort definition excluded patients undergoing cardiac - or craniotomy surgical procedures.”

We also highlighted our retrospective design as a clear limitation with “A prospective study that validates the predictions of our models also focusing on a clinical assessment regarding the Nu-DESC would be beneficial towards a clinical application.”

We addressed potential documentation biases by “We rather ensured that extracted variables reflected our front-end systems. Our univariate analysis and prediction models based on clinical data that could potentially comprise extreme values (e.g. body temperature of 44.3 °C) due to flawed documentations. We believe that our models benefited from these noisy data by using robust MWU statistics and robust feature aggregations - like percentiles - increasing model’s applicability. However, univariate results must be interpreted carefully since no cohort matching – e.g., for reducing cofounding bias in a case -control study – was done”

10. The conclusion could address future research directions more directly and how these findings could influence clinical practice or policy.

RESPONSE: Thank you for your comment. We enhanced our Conclusion based on your suggestion by “We presented the clinical impact of one model configuration outbalancing patient safety and overtreatment. Results could guide decision makers that are eager to explore machine learning for mitigating POD risks.”

Reviewer #3: In the author summary paragraph, I suggest rewording and defining “mentally disturbed”. For example, consider “Many patients who experience POD suffer from chronic mental health disorders…”. Also consider rewording “With our study, we want to detect POD before...” to “With our study, we want detect suspected POD”.

RESPONSE: Thank you very much for suggesting rephrasing our Author Summary. We included your rewordings as “Many patients who experience POD suffer from chronic mental disorders causing pressure on related family members, clinicians, and the health system. With our study, we want to detect suspected POD before onset trying to give decision support to health professionals.”

“Vulnerable patients could be transferred to delirium wards mitigating the risk of severe outcomes such as permanent cognitive decline.” Are there delirium wards in Germany? Consider saying “transferred to a higher level of care…” Also question the use of “permanent cognitive decline”. Consider using “long-term cognitive decline”.

RESPONSE: We really appreciate your suggestions that increase the clinical correctness of our phrases. According to your comment, we wrote, “Vulnerable patients could be transferred to a higher level of care mitigating the risk of severe outcomes such as long-term cognitive decline.” As we know, German medical centers rarely reserve additional delirium wards for vulnerable patients. At our clinical institution, we have implemented modified ICUs aiming to reduce delirium severity by light-concepts and noise reduction [1].

[1] Spies, C., Piazena, H., Deja, M., Wernecke, K. D., Willemeit, T., Luetz, A., & ICU Design Working Group. (2024). Modification in ICU Design May Affect Delirium and Circadian Melatonin: A Proof of Concept Pilot Study. *Critical Care Medicine*, *52*(4), e182-e192.

While the etiology of POD is difficult to prove conclusively, Li et al. (2022) make the case that while surgical stress can lead to local inflammation and immune activation, it can also lead to a systematic cascade of inflammatory signaling molecules which induce, maintain, and aggravate neuroinflammation and subsequent POD, especially in the elderly population. This makes sense as Andronie-Cioara et al. (2023) report that aging is associated “with a chronic inflammatory state both in the periphery and in the central nervous system”. Therefore the activation of the inflammatory cascade during a surgical procedure in patients with chronic neuroinflammation is a viable explanation for POD in elderly and in patients with other underlying chronic inflammatory conditions.

Li Z, Zhu Y, Kang Y, Qin S, Chai J. Neuroinflammation as the Underlying Mechanism of Postoperative Cognitive Dysfunction and Therapeutic Strategies. Front Cell Neurosci. 2022;16:843069. Published 2022 Mar 28. doi:10.3389/fncel.2022.843069

Andronie-Cioara FL, Ardelean AI, Nistor-Cseppento CD, et al. Molecular Mechanisms of Neuroinflammation in Aging and Alzheimer's Disease Progression. Int J Mol Sci. 2023;24(3):1869. Published 2023 Jan 18. doi:10.3390/ijms24031869

RESPONSE: We appreciate your valuable clinical knowledge with respect to possible physiological pathways of POD. According to your extensive explanations, we enhanced our Introduction by “While the etiology of POD is difficult to prove conclusively, authors investigated surgical stress as a potential cause for neuro inflammation that could subsequently result in POD, especially in the elderly population” and cited your references.

We agree that POD is a highly complex clinical phenomenon. In our study, we aimed to develop a data-driven prediction model rather than explaining the bio-chemical mechanisms that could cause POD. Clinical experts and health professionals should interpret our model results including the predictors that we have presented.

What does ASA status in the abstract refer to? Is it American Society of Anesthesiologists (ASA) Physical Status Classification System? What is “OPS” surgical procedure history (page 10)? All acronyms need to be spelled out prior to use.

RESPONSE: We totally agree that all acronyms need to be spelled out prior to use. According to your comments, we explained ASA status in the Abstract as “Significant univariate impact factors were the preoperative ASA status (American Society of Anesthesiologists physical status classification system) status, the intraoperative amount of given remifentanil, and the postoperative Aldrete score.” Additionally, we added a novel chapter in the main manuscript that described our feature encodings and explained ICD and OPS.

“Demographic characteristics – e.g., age, weight – were included without any extra feature encoding and assigned to the entire hospital stay (TI). The history of an International Classification of Diseases (ICD) code was summarized as the number of respective diagnoses from previous hospital stays of the same patient and assigned to TI. Hospitalization information and life support procedures – e.g., hospitalization durations, surgery durations, and ventilation durations – were calculated for each time phase (T1-T3) individually (…)

Information from German operation and procedure (OPS) codes were used to one-hot encode different types of operation procedures – e.g., procedures on the nervous system. These classes were considered to be fixed before each hospital stay (TI). Drug classes – e.g., benzodiazepines, opioids – and types of anesthesia procedures – e.g., preoxygenation – were one-hot encoded as time-variant variables. We encoded ICD diagnoses according to their system entry time during a hospital stay as a binary variable, separately for each time phase. Concrete ICD and well as OPS codes are provided in Extended Table 2 in Multimedia Appendix 1.”

In Table in the supplement the lower bounds for GCS is listed as 0. The lowest possible GCS score is 3. Are 0 values missing values? What imputation methods were used and how did the researchers determine if missingness was structurally missing, missing completely at random (MCAR), missing at random, or missing not at random?

RESPONSE: Thank you for sharing your sharp observation. We did not include GCS in our final feature set since the variable was not available for at least 10% of the patients. We explain, “Based on literature review and clinical expertise, a total of 549 clinical variables were identified with respect to T1-T3 and TI in our source systems. From these variables, 375 were available for at least 10% of the included patients.” However, we agree that the GCS scale has a minimum of 3 and changed our lower bound.

We conducted our univariate statistical analyses with robust Mann-Whitney U (MWU) [1] statistics that did not require imputing missing values. Before feeding data into our machine learning models, we standardized data points by z-transformation for harmonizing different scales. Z-transformation [2,3], commonly used in machine learning, divides every deviation from the mean for one element in a series of values by the standard deviation. We used means for imputing unobserved values that were also part of the z-transformation. By doing so, the model could directly learn the deviation of single values from the mean.

Thanks for requesting clarification of our missingness assumptions. We think that we cannot test our complete data to specify the concrete missingness pattern. In our opinion, especially the highly complex missing not at random (MNAR) case is in impossible to prove, since the reasons for the absence of data points is not included in the observed data [4]. However, we included a separate analysis “Additionally we encoded missingness with binary indicators. Extended Figure 2, that is included in Appendix 2, shows correlations between missing indicator variables assuming that our data did not follow a missing completely at random (MCAR) pattern.”

Additionally, we concluded in our Discussion that our best applicable model could learn from encoded availability information. “We retrieved MLP input weights (w) for 441 features including missing indicators recorded during T12. We identified age (w=0.907), history of delirium (w=0.784), and history of hypertension (w=0.631) as important model inputs. (...) We observed elevated model weights regarding the binary missing indicator for intraoperative pulse (w=0.526) and sedation depth (w=0.379). Observations suggest that POD predictions can benefit from encoded availability information.”

[1] Michael P. Fay, Michael A. Proschan "Wilcoxon-Mann-Whitney or t-test? On assumptions for hypothesis tests and multiple interpretations of decision rules," Statistics Surveys, Statist. Surv. 4(none), 1-39, (2010), 10.1214/09-SS051

[2] P. Sharma and J. Singh, "Machine Learning Based Effort Estimation Using Standardization," 2018 International Conference on Computing, Power and Communication Technologies (GUCON), Greater Noida, India, 2018, pp. 716-720, doi: 10.1109/GUCON.2018.8674908.

[3] Mohamad, I. B., & Usman, D. (2013). Standardization and its effects on K-means clustering algorithm. Research Journal of Applied Sciences, Engineering and Technology, 6(17), 3299-3303

[4] Haneuse S, Arterburn D, Daniels MJ. Assessing Missing Data Assumptions in EHR-Based Studies: A Complex and Underappreciated Task. JAMA Netw Open. 2021;4(2):e210184. doi:10.1001/jamanetworkopen.2021.0184

I also question the vital sign lower bounds (all were 0 except temperature) and even the upper bounds were not conducive with life. For example, a temperature of 10 Celsius (50 Fahrenheit) or 50 Celsius (122 Fahrenheit) are not conducive with life and are likely artifacts. How did researchers manage these artifacts. The same goes for measurements of 0 for respiratory rate, heart rate, blood pressure… My assumption is that these were either missing values or artifacts. How were these managed? I have never seen a patient on a tidal volume of 10,000 ml. I have similar concerns with lower/upper limits in other variables in this table.

Description of Table 1 in the supplementary Excel Spreadsheet: Extended Table 1: Hard rules in terms of ranges from valid lower - to upper bounds for data preprocessing. Values that do not fall into the valid range were imputed with the mean calculated on the training set.

In the Extended Table 3 of this same spreadsheet seems to report high rates of missingness as well as some min and max values not conducive with life. See example below.

feature	set	domain	unit	tl	missingness_rate	min	max

body temperature	train	Vital Signs	°C	2	0.44	10.90	45.40

body temperature	train	Vital Signs	°C	1	0.96	10.30	44.30

body temperature	train	Vital Signs	°C	3	0.99	12.90	39.80

body temperature	test	Vital Signs	°C	2	0.45	16.20	39.90

body temperature	test	Vital Signs	°C	1	0.95	10.20	39.90

body temperature	test	Vital Signs	°C	3	0.99	18.30	38.30

RESPONSE: Thank you for expressing your concerns regarding potential outliers and artifacts. We totally agree that retrospectively extracted electronic health records could comprise values that might not be conducive with life. Oftentimes, we could not differentiate between an erroneous recorded artifact and a real physiological edge case (e.g. heart rate of 220 due to movement). Additionally, we believe that a rigorous elimination of artefacts renders the models less useful in practice, given that artefacts are a reality in the data. The model must behave gracefully, even with numerical outliers. Instead of discarding these values, potentially resulting in losing predictive information, we applied robust methods that can handle extreme values inherently.

“For numerical variables, valid thresholds (lower and upper bounds) were applied to remove impossible values (e.g., negative oxygen saturation) rather than omitting physiological edge cases (e.g., oxygen saturation at 65%) (see Extended Table 1 in Appendix 1). Hence, extreme values were kept initially that might contribute to the predictive performance. We preferred preserving raw data instead of heavy preprocessing. The application of robust feature aggregations increased the applicability of our methods. Multiple values for one surgery recorded during one time phase were aggregated as described in the next section.”

Firstly, we aggregated multiple values recorded during one surgery with robust statistics like 10^th^, 50^th^, and 90^th^ percentiles. Afterwards, we applied the robust non-parametric MWU test statistics for retrieving univariate results [1]. The MWU does not assume normal distributed values and calculates statistics over ranks of data points. Hence, the test is capable of handling outliers and skewed value distributions. We selected our robustly aggregated features for training machine learning models. By avoiding heavy preprocessing, we increased the applicability of our models capable of ingesting uncertain data. However, we repeatedly advice that presented results should be used to explain model predictors instead of directly affecting clinical practice.

In the Discussion, we include, “Due to the vast amount of records, there was no chance of ensuring clinical correctness for all extracted EHRs. We rather ensured that extracted variables reflected our front-end systems. Our univariate analysis and prediction models based on clinical data that could potentially comprise extreme values (e.g. body temperature of 44.3 °C) due to flawed documentations. We believe that our models benefited from these noisy data by using robust feature aggregations - like percentiles - increasing model’s applicability. However, univariate statistical results must be clinically interpreted carefully. No cohort matching – e.g., for reducing cofounding bias in a case -control study – was done.” and “Since we applied L1-norm regularization on the first MLP layer, we could retrieve model input weights. We believe that these findings should contribute to further clinical validation steps instead of directly impacting clinical practice due to the high complexity of ML models”.

We changed our descriptive statistics in Appendix 1 in Extended Table 3 accordingly to “Based on literature review and clinical expertise, a total of 549 clinical variables were identified with respect to T1-T3 and TI in our source systems. From these variables, 375 were available for at least 10% of the included patients”. In the current revision, we solely include features that were potentially used by our models. Since we observed high missing rates for body temperature during the pre- and postoperative phase, we describe this feature just for the intraoperative phase.

[1] Michael P. Fay, Michael A. Proschan "Wilcoxon-Mann-Whitney or t-test? On assumptions for hypothesis tests and multiple interpretations of decision rules," Statistics Surveys, Statist. Surv. 4(none), 1-39, (2010), 10.1214/09-SS051

Table 1: Snipped Extended Table 3

| **feature** | **set** | **missingness** | **min** | **max** | **mean** | **std** | **10_perc** | **25_perc** | **50_perc** | **75_perc** | **90_perc** |
| --- | --- | --- | --- | --- | --- | --- | --- | --- | --- | --- | --- |
| body temperature | test | 0.448 | 16.2 | 39.9 | 36.25 | 0.890 | 35.5 | 35.9 | 36.3 | 36.7 | 37 |
| body temperature | train | 0.436 | 10.9 | 45.4 | 36.24 | 0.907 | 35.5 | 35.9 | 36.3 | 36.7 | 37 |

In Table 1 (above), we include 10^th^ and 90^th^ percentiles for reporting purposes calculated on all feature values without aggregation (potentially more than one value per surgery and patient). These percentiles were at 35.5 and 37°C for the measured body temperature across test and training sets, respectively. After aggregating multiple values per surgery, we saw the following (Table 2) summary statistics for the 50^th^ percentile of body temperature (body_temperature_p0.5):

| **feature** | **set** | **mean** | **std** | **min** | **max** | **10_perc** | **25_perc** | **50_perc** | **75_perc** | **90_perc** |
| --- | --- | --- | --- | --- | --- | --- | --- | --- | --- | --- |
| body_temperature_p0.5 | train | 36.2 | 0.71 | 20.3 | 39.5 | 35.6 | 35.9 | 36.2 | 36.6 | 36.9 |
| body_temperature_p0.5 | test | 36.2 | 0.58 | 32 | 39.9 | 35.55 | 35.9 | 36.2 | 36.6 | 36.95 |

Table 2: Snippet Extended Table 4

Although these descriptive statistics for median-aggregated temperature values seemed to be more conducive with life, we highlighted as mentioned before, that results must clinically interpreted carefully. We deeply believe that researchers who have gained a lot clinical expertise - like you – conducting POD research can put our findings into perspective.

[1] Michael P. Fay, Michael A. Proschan "Wilcoxon-Mann-Whitney or t-test? On assumptions for hypothesis tests and multiple interpretations of decision rules," Statistics Surveys, Statist. Surv. 4(none), 1-39, (2010), 10.1214/09-SS051

Lastly, there was limited demographic information (only included age, BMI, and gender) so it is difficult to determine generalizability.

Unfortunately, we do not have additional demographic information like race or ethnicity, social status, education, or native language in our dataset. These features are not routinely collected in Germany.

We reported all extracted and aggregated feature values for our study cohort.

The researchers need to better explain how they prepared data for analysis. Did homologation occur e.g. checking the mapping of the data for correctness, consistency, and possible corruption after the ETL process? Did data corruption occur during the ETL process (resulting in deletion or modification of information, inclusion of extraneous or confounding data, and misinterpretation of the clinical meaning of data)? If those steps were not feasible then how were the meanings of variables determined/managed?

RESPONSE: Thank you for requesting more additional information about our ETL-pipelines. According to your comments, we enhanced our section Feature Extraction and Preprocessing.

“EHRs might suffer from integrity issues due to distributed CIS. Thus, extraction, transformation, and loading (ETL) scripts were refined until there were no discrepancies with the front-end for a sample of 50 surgeries. We systematically checked these sample data for correctness and consistency with clinical experts. We integrated variables across source systems and harmonized different naming conventions as well as units. The data warehousing team at our institution provided technical documentation to understand the CIS e.g., the versioning mechanism, calculation of metrics, and data schema descriptions. Reverse engineering was applied to reconstruct data that was displayed in our front-end application.”

--------------------

<font color = "black">6. PLOS authors have the option to publish the peer review history of their article (<a href="https://journals.plos.org/digitalhealth/s/editorial-and-peer-review-process#loc-peer-review-history" target="_blank">what does this mean?</a>). If published, this will include your full peer review and any attached files.<br><br>

<b>Do you want your identity to be public for this peer review?</b> If you choose “no”, your identity will remain anonymous but your review may still be made public.<br><br>

For information about this choice, including consent withdrawal, please see our <a href="https://www.plos.org/privacy-policy" target="_blank">Privacy Policy</a>.</font>

Reviewer #1: No

Reviewer #2: No

Reviewer #3: No

--------------------
